# Cytomegalovirus infection in immunocompetent critically ill adults: literature review

**DOI:** 10.1186/s13613-016-0207-8

**Published:** 2016-11-03

**Authors:** Awad Al-Omari, Fadi Aljamaan, Waleed Alhazzani, Samer Salih, Yaseen Arabi

**Affiliations:** 1 Critical Care and Infection Control Department, Dr. Sulaiman Al Habib Medical Group, Riyadh, Saudi Arabia; 2 AlFaisal University, Riyadh, Saudi Arabia; 3 Intensive Care Department, King Khalid University Hospital, King Saud University Medical City, King Saud University, Riyadh, Saudi Arabia; 4 Department of Medicine, McMaster University, Hamilton, Canada; 5 Department of Internal Medicine, Dr. Sulaiman Al Habib Medical Group, Riyadh, Saudi Arabia; 6 Intensive Care Department, King Abdullah International Medical Research Center, King Saud Bin Abdulaziz University for Health Sciences, Riyadh, Saudi Arabia

**Keywords:** Cytomegalovirus, Infection, Immunocompetent, Critical illness

## Abstract

Cytomegalovirus (CMV) infection is increasingly recognized in critically ill immunocompetent patients. Some studies have demonstrated an association between CMV disease and increased mortality rates, prolonged intensive care unit and hospital length of stay, prolonged mechanical ventilation, and nosocomial infections. However, there is a considerable controversy whether such association represents a causal relationship between CMV disease and unfavorable outcomes or just a marker of the severity of the critical illness. Detection of CMV using polymerase chain reaction and CMV antigenemia is the standard diagnostic approach. CMV may have variety of clinical manifestations reflecting the involvement of different organ systems. Treatment of CMV in critical care is challenging due to diagnostic challenge and drug toxicity, and building predictive model for CMV disease in critical care setting would be promising to identify patients at risk and starting prophylactic therapy. Our objective was to broadly review the current literature on the prevalence and incidence, clinical manifestations, potential limitations of different diagnostic modalities, prognosis, and therapeutic options of CMV disease in critically ill patients.

## Background

CMV disease is usually a disease acquired by adolescence and follows a benign course, while it might reactivate in patients with immune suppression and associated with high mortality and morbidity.

There is growing evidence that critically ill immunocompetent patients can develop CMV disease. Studies have described CMV infection in immunocompetent surgical, septic, burn, or trauma critically ill patients [1, 2]. Some studies have demonstrated an association between CMV disease and increased mortality rates, prolonged (ICU) and hospital length of stay, prolonged mechanical ventilation, and increased rate of nosocomial infections [2, 3]. However, there is a considerable controversy whether such association represents a causal relationship between CMV disease and unfavorable outcomes, or whether CMV disease only represents a marker for severe illness that carries poor clinical outcomes.

The objective of this review is to examine the up-to-date literature regarding CMV disease in critically ill patients. We will review the studies that assessed prevalence and incidence of CMV disease refuting the differences among them leading to different results, clinical manifestations, pathogenesis, potential limitations of different diagnostic modalities, association with outcomes, and treatment options of CMV disease in critically ill patients.

## Definitions

Primary CMV infection usually occurs during childhood and early adolescence and is usually asymptomatic or mild and self-limiting disease in immunocompetent patients. After the resolution of acute infection, CMV establishes latent phase mainly within leukocytes, namely mononuclear cells, and this stage is diagnosed with a positive anti-CMV IgG serology (seropositivity) and is characterized by maintenance of the viral genome in the absence of production of lytic infectious virions but with the ability of the viral genome to reactivate under certain conditions [3].

CMV literature focused on immunocompromized population like transplant and AIDS populations; therefore, CMV disease statuses and definitions were based on that literature which defined CMV infection as detection of CMV genetic or molecular material in the patients’ serum or body fluids, indicating active replication of the virus. CMV infection according to that literature can occur as a result of reactivation of latent CMV or reinfection with an exogenous strain. Recurrent infection in immunocompromized patients is the detection of CMV genetic or molecular material in a patient who had previous documented infection and who has not had virus detected for an interval of at least 4 weeks during active surveillance [4]. Recurrent infection could be due to reactivation (if the detected strain is the same as the primary infecting strain) which is the most common mechanism of acquiring the disease in adulthood, especially in immunocompromized population (seropositivity of the donor or recipient has been recognized as the most likely mechanism of acquiring the disease after organ transplant; therefore, it is the recommended strategy for starting CMV prophylactic treatment across most guidelines) [5–7], or new infection (different strains) [4]. Additionally, de novo primary infection may occur in adults but is rare. However, the distinction between the three scenarios, reactivation, new strain infection, and de novo primary infection, is difficult in practice, unless the patient is followed for a long time or the infecting source CMV genetic strains have been characterized.

CMV disease in critically ill adults will probably fall under one of these three mechanisms. Meta-analysis done by Osawa and Singh [8] has shown that CMV-seronegative critically ill patients rarely developed CMV infection across most studies, suggesting that reactivation is the most likely mechanism of CMV disease in this population. However, the other two mechanisms may be responsible for the infection in some cases.

CMV end-organ disease has been defined as evidence of virus replication associated with clinical manifestations due to viremia or the invasion of organs such as the lungs, bone marrow, and colon [4].

The scope of this article is to examine CMV disease literature in critically ill overtly immunocompetent patients.

In summary, CMV is usually acquired in childhood, and the most common mechanism of the disease in adulthood is reactivation of latent virus whether in immunocompromized or critically ill population, followed by rare incidence of recurrent new or de novo new infection.

## Virus genome, pathogenesis, and virulence

CMV is the most common member of the herpes viruses to infect humans. Its double-stranded linear DNA duplex contains 165 genes that encode viral proteins that mimic and interact with human cellular proteins and are related to its virulence and latency.

CMV is maintained in a latent or low production state within monocytes mainly and dendritic cells (DC). They do not usually express viral genes in significant number due to the robust CD8+ cytotoxic T lymphocyte response and T memory cells. In immunocompetent individuals, asymptomatic viral shedding may be detectable in the saliva or urine; however, cell-mediated host immune responses prevent the development of overt CMV disease [3].

CMV genome is detected within early progenitor myeloid CD34+ cells, and monocytes and (DC) differentiation will be the only lineage that will pass the CMV genome. Viral expression is closely related to expression of immediate early (IE) genes within monocytes and (DC), but expression of infectious (lytic) virions only happens within (DC), a process regulated by major IE enhancer promoter (MIEP) which is normally under repressors closely related to other factors and cytokines within the cells [3].

CMV disease occurs as a result of immunosuppression associated with critical illness. A landmark study by Clari showed that CMV infection in critically ill patients was consistently associated with undetectable IFN-γ T cell responses within the first 2 days of admission to the ICU, and that viral load was inversely related to IFN-γ T cell responses [9]. A study by Venet showed that septic patients display immune system paralysis, manifesting as reduced Th1 B cell function, increased IL-10 production (anti-inflammatory), and global lymphopenia affecting natural killer cells (NK) specifically quantitatively and most importantly qualitatively related to their interferon production, which is pivotal in attacking CMV-infected cells [10, 11]. CMV disease has also been linked to the cytokine storm associated with critical illness, specifically tumor necrosis factor alpha that activates nuclear factor κB, which enhances the replication of the dormant CMV DNA inside leukocytes, while enhancing the production of cytokines and other proteins [12]. In animal models with bacterial sepsis, Toll-like receptor 4 signaling and secreted inflammatory cytokines all have been found to be potential triggers for reactivation of latent CMV in immunocompetent mice lungs [13].

Under such critical conditions observed during sepsis, burns, trauma, or major surgery, the CMV genes are expressed and viral replication is initiated [14, 15], invading cells in the lung, kidney, liver, bone marrow, and intestine [3], and exerts direct cytotoxic effects.

In addition, when CMV starts to replicate within leukocytes, it has inherent escape mechanisms from the host immune system, and the CMV unique short (US 2, 3, 6, 10, and 11) proteins down-regulate the surface expression levels of HLA-1 and HLA-2 on leukocytes that mark these cells to be attacked by CD8+ T lymphocytes [16].

Other CMV genes encode structural proteins, such as the matrix protein pp65, that is involved in down-regulation of HLA-1 leukocytes surface markers and used as a target of antigen detection immune assays [17].

In addition, virulence is also related to boosting immune response causing more tissue damage, and this is due to homology of some of CMV proteins to inflammatory cytokines as human tumor necrosis factor alpha receptor and CXC chemokine such as interleukin-8 [18].

Animal model studies suggested that the outcome after reactivation might be determined by the viral load of the original infection, which correlates with the number of CMV-specific T memory cells, i.e., immunological responses (higher IgG levels), CD-8 cytotoxic cells, and hence inflated immune response during reactivation, and this might be a promising marker to predict the outcome in patients with reactivation [13, 19].

In addition, the virus has an immunosuppressive effect via its modulatory effect on cytokine production, which may enhance the susceptibility to secondary bacterial and fungal infections. CMV also has a direct suppressive effect on the bone marrow.

Some CMV proteins are also related to antiviral mechanisms, including the viral DNA polymerase UL54 and UL97, which encodes a protein phosphotransferase enzyme that phosphorylates the antiviral drug ganciclovir, an essential activation step required for its inhibitory effect on CMV DNA replication [20–22].

In summary, CMV has a complicated genome that allows the virus to go into latent state and facilitate its evasion from immune response and homology to human chemokines, all of which is involved in its pathogenesis.

## Risk factors

Several factors have been identified to be associated with CMV disease in critically ill patients (Table 1), including requirement of mechanical ventilation on admission [8], an inflammatory status like sepsis [23–25]. Other reports have linked CMV disease to steroid use, but this finding has not yet been confirmed [26–28]. In addition, catecholamines surge associated with critical illness has been linked to CMV disease in myocardial infarction patients [25]. Several studies failed to show a correlation between age and CMV infection [1, 24, 27, 29, 30], while the reported association with gender is inconsistent across the literature [1, 2, 24, 27–29, 31]. Interestingly, higher disease severity scores such as the acute physiology and chronic health evaluation (APACHE) II [1, 27, 29] and sepsis-related organ failure assessment (SOFA) [30] scores have not been found to be associated with increased risk of CMV disease. Active malignancy has not been shown to be a risk factor for CMV disease in critically ill patients [2, 24, 28].

**Table 1:** Risk factors for CMV disease in critical care setting and their strength of association

Risk factor	Strength of association
Immune compromised	Strong association [49]
Age	No evidence [8]
Gender	Inconsistent data [8]
Mechanical ventilation	Strong association [1, 8]
Sepsis	Strong association [8, 47]
Corticosteroids use	Weak evidence [27]
Blood transfusion	Weak association [1, 27, 28]
Disease severity scores	No association [1, 24, 27–31]
Active malignancy	No association [2, 24, 28]
Stress (catecholamines surge)	Weak association [25]

Blood transfusion, especially in the first 24 h of critical illness, has been shown to be an important risk factor for CMV disease [1], which has been linked to the timing and the amount of blood transfused, although the mechanisms involved are unclear. Contamination of transfused blood with CMV-infected leukocytes may be responsible for some cases, especially in cases where the blood bank does not perform leukocyte depletion pre-transfusion [32, 33], even though leukocyte-depleted blood is not completely clear of leukocytes; hence, its use does not completely eliminate the risk of CMV infection. In addition, the immunomodulatory effect of transfusion may also predispose patients to CMV reactivation.

In summary, mechanical ventilation and blood transfusion in the ICU have been associated with the development of CMV disease, but no association with severity scores was found.

### Epidemiology

Several epidemiologic studies assessed the incidence of CMV disease in critically ill patients (Table 2), and these studies used different methodologies that lead to variation in the observed incidence of infection ranging from 0% to as high as 98%. This inconsistency in the results could be explained by many factors as variation in the definition of CMV disease (old studies considered seropositivity as evidence of CMV disease, while others used newer technologies as PCR and antigen detection), variation in inclusion criteria as some studies included only seropositive patients and hence assessed only reactivation rate of CMV rather than CMV infection rate which might be slightly higher than reactivation by including seronegative patients developing new infection in addition to reactivation, all that in addition to variation in studied populations that ranged from all ICU patients to specific population such as septic, surgical, burn, or post-cardiac surgery patients that might have different risks of CMV disease.

**Table 2:** Summary of studies assessing CMV disease in ICU patients

Year of publication	Study	Study design	Patient population	Patients no.	Method and specimen	Frequency of monitoring	Incidence (%)	Mortality (%)	
								CMV positive^a^	CMV negative^b^
1990	Domart et al. [31]	Prospective, single center	Mediastinitis, post-cardiac surgery Patients in whom viral cultures could be done within 10 days of surgical debridement	115	Serology for CMV IgG Viral culture blood and urine. Positive cases: positive CMV culture	Within first 10 days of debridement then every 3 weeks	25	55*	37*
1994	Docke et al. [50]	Unidentified	Septic patients	60	1—CMV culture in blood. 2—Immunocytological detection of CMV antigens in blood. 3—PCR amplification in blood. 4—TNF and interleukin six assays. Positive cases: any positive PCR or culture or antigen detection	Once	97.7	NA	
1996	Stephan et al. [51]	Prospective, single center	Mechanically ventilated	23	Culture, PCR Blood and BAL	Unclear	0	0	
1996	Papazian et al. [39]	Retrospective Single center	Patients with ventilator-associated pneumonia	86	Histopathology Autopsies and open lung biopsy	Once 22.4 ± 8.8 days after admission to the intensive care unit	29	NA	NA
1998	Kutza et al. [29]	Prospective, single center	SICU Septic patients with positive CMV IgG serology	34	CMV IgG serology CMV antigenemia CMV DNA PCR in blood Serum cytokines: TNF alpha, IL-1b, and IL-6 measured by ELISAs. Positive cases: positive CMV antigenemia and/or CMV DNA PCR in blood	1—Blood samples were collected on day 1 of sepsis, then twice weekly during ICU stay, then once weekly after release from the ICU, until recovery or death	32	73.9*	63.6*
1998	Cook et al. [27]	Prospective, case–control single center	Postoperative general SICU. Septic patients with no identifiable bacterial or fungal source. Positive BAL or blood or sputum CMV culture	12	CMV and HSV viral culture from BAL, blood or sputum. All cases included had positive CMV culture	CMV was cultured in diploid human foreskin fibroblast culture for 6 weeks Plasma was evaluated by shell vial technique to IE antigen at 24 h. HSV cultures	100	65^α^	35^α^
2001	Heininger et al. [24]	Prospective, single center	SICU Patients with SAPS II >40 and positive serology	56	CMV DNA PCR and/or viral culture Leukocytes, plasma, and lower respiratory tract secretions Positive cases: positive PCR	Once weekly	35.6	55*	36.2*
2002	Razonable et al. [52]	Prospective Case–control Single center	Mixed ICU	120	CMV and HHV-6 and HHV-7 DNA by PCR in blood Positive cases: positive PCR	4th days after ICU admission	<1	NA	
2003	Cook et al. [23]	Prospective Single center	SICU Patients staying for 5 days or more in SICU	104	Serology in blood CMV and HSV cultures in blood and sputum Positive cases: positive CMV culture	Once weekly until discharge from SICU	9.6	50*	26.5*
2005	Jaber et al. [28]	Retrospective, matched case–control, single center	Medical, surgical, and transplant unit Non-immune-compromised patients	237	PP65 antigen Blood Positive cases: positive CMV antigenemia	1—Clinical judgment. 2—The diagnosis of CMV antigenemia was defined by a positive CMV pp65 antigenemia assay result	16.8	50^α^	28^α^
2006	von Muller et al. [53]	Prospective observational single center	Anesthesiological intensive care unit. Non-immunocompromized CMV IgG-seropositive patients with septic shock with ICU stay for at least 7 days	23	Serology CMV antigenemia Blood. CMV reactive T helper 1 lymphocytes Positive cases: positive CMV antigenemia	Twice during 1st week then weekly until discharge Positive cases: positive antigenemia	30.4	57*	38*
2008	Ziemann et al. [2]	Retrospective, single center	Mixed ICU Patients treated for at least 14 days in ICU	99	CMV DNA PCR Blood. Positive cases: at least two positive samples	Based on the decision of the treating team	35^∑^	28.6^α^	10.9^α^
2008	Limaye et al. [1]	Prospective, multicenter	Mixed ICU, positive anti-CMV IgG	120	PCR Blood Positive cases: positive blood CMV PCR	Thrice weekly until death or discharge	33	CMV disease was significantly associated with death or continued hospitalization after 30 days	
2009	Chiche et al. [26]	Prospective, single center	MICU Ventilated patients for at least 2 days	242	CMV serology and antigenemia. CMV culture on BAL if VAP suspected, antigenemia on the day of BAL. Other organ Virologic studies based on clinical suspicion. Positive cases: positive antigenemia or BAL CMV culture	Within 48 h of ICU admission and once weekly until discharge	16.1	54*	37*
2010	Chilet et al. [54]	Prospective observational Single center	Anesthesiological ICU Single center CMV-seropositive, ICU stay longer than 5 days	53	CMV PCR Tracheal aspirate and plasma Plasma tumor necrosis factor alpha	Once a week	39.7	61*	46*
2010	Smith et al. [55]	Prospective single center	Tertiary ICU Mechanically ventilated patients	174	Respiratory secretions CMV PCR Thrice weekly		19	NA	
2011	Iahangard	Prospective	ICU CMV-seropositive patients	132	CMV IgG serology PCR in blood	Weekly	34	The overall mortality rate associated with active CMV infection was 1.93 times higher than that without CMV infection	
2011	Bordes et al. [56]	Prospective Single center	Severe burn unit Burn surface area more than 15%	29	CMV DNA Blood	First sample on admission then one to twice weekly until discharge	55	33*	20*
2011	Uegaki	Retrospective Single center	ICU	67	CMV antigenemia	After 7 days of admission if intensivists suspected CMV	52	NA	
2011	Heininger et al. [38]	Prospective, observational, single center	Severe sepsis, positive anti-CMV IgG	86	Viral culture from tracheal secretions. Qualitative CMV DNA PCR from leukocytes, plasma, tracheal secretions Quantitative CMV DNA PCR from positive plasma and tracheal secretions HSV DNA PCR from tracheal secretions Positive cases: positive PCR from any source	Once weekly until discharge from hospital or death	40.7	37.1*	35.3*
2012	De Vlieger et al. [57]	Prospective, MICU and SICU, single center (Van Den Berge study)	Admitted for at least 3 days in ICU Mechanically ventilated in SICU MICU regardless of mechanical ventilation	1504	CMV IgG serology	Within first 2 days of admission	64	19.2*	17.1*
2012	Chiche et al. [10]	Prospective Single center	MICU CMV-seropositive patients	82	CMV serology CMV antigenemia Lymphocyte subset Interferon-γ	Within 48 h of admission then once weekly until discharge	27	40*	13.3*
2012	Coisel et al. [37]	Prospective single center	MICU Mechanically ventilated suspected of having pneumonia 10% were previously immunocompromized	93	1—Serology (IgM and IgG) for cytomegalovirus (CMV) and herpes simplex (HSV) using (ELISA) Blood PCR was not routinely done 2—CMV antigen detection in blood 3—CMV and HSV DNA PCR on BAL Positive cases: positive CMV PCR in BAL or positive antigenemia or positive IgM	Within 12 h of pneumonia diagnosis	24	55^α^	20^α^
2013	Clari et al. [9]	Prospective single center	SICU Mechanically ventilated patients CMV seropositive	31	CMV serology at baseline CMV-specific interferon-γ-producing CD8, CD4 T lymphocytes CMV PCR in blood and tracheal aspirate	2–8 samples per patient were analyzed for T lymphocytes subset and CMV PCR	54.8	47*	35*
2014	Ishioka et al. [58]	Prospective Single center	Cardiac ICU Post-surgery	100	CMV PP65 antigenemia Serum	On admission, day 7 and day 14	4	0*	1*
2014	Osman	Prospective Single center	Respiratory and geriatric ICU	51	CMV PCR Blood	NA	68.6	74.3^α^	31.3^α^
2014	Walton et al. [59]	Prospective Single center	MICU and SICU Critically ill septic and non-septic patients versus healthy volunteers	560	CMV IgG CMV PCR Blood	Daily Starting within 24–72 h of admission	24.2	Significant increase in 90 days mortality in CMV-positive patients compared to CMV-negative ones	
2015	Ong et al. [40]	Multicenter prospective cohort	Mixed ICU (tertiary care referral centers) ARDS patients on mechanical ventilation for at least 4 days	271	CMV IgG if positive CMV PCR in blood	Weekly for maximum 30 days	27	46^α^	28^α^
2015	Frantzeskaki et al. [12]	Prospective, observational two centers	Mixed ICU, mechanically ventilated, seropositive anti-CMV IgG	80	CMV DNA PCR Blood	At admission, then weekly until day 28 or discharge from ICU or death	13.75	18*	22*
2015	Ong et al. [60]	Multicenter prospective cohort	Mixed ICU (tertiary care referral centers) ARDS patients on mechanical ventilation for at least 4 days	209	CMV IgG between days 5 and 14 of admission If positive CMV DNA PCR in plasma	CMV DNA PCR on day 14 or ICU discharge date (whichever first) then weekly	26	28*^,δ^	16*

Positive serology: positive anti-CMV IgG or IgM

*ELISA* enzyme-linked immunosorbent assay, *PCR* polymerase chain reaction, *CMV* cytomegalovirus, *ARDS* acute respiratory distress syndrome, *BAL* bronchoalveolar lavage, *MICU* medical intensive care unit, *SICU* surgical intensive care unit, *SAPS* simplified acute physiology score, *ICU* intensive care unit, *ELISA* enzyme-linked immunosorbent assay

^a^CMV positive based on study methodology

^b^CMV negative based on study methodology

* Nonsignificant difference in mortality

^α^Statistically significant difference in mortality between groups

^δ^24% in seropositive without reactivation

^∑^Considered infected if at least two positive results

Variation in diagnostic methods used including selecting different specimens, some used serum, while others used urine, saliva, or bronchoalveolar lavage samples, tests were performed at different points in the ICU stay and testing time and frequency was not standardized in all studies as some left it up to the judgment of the treating team. Recent studies showed that CMV disease typically occurs within the first 2 weeks of critical illness. A study performed by Kalil and Florescu [34] showed that the diagnosis rate of CMV infection increased significantly (from 1 to 21%) when patients who spent five or more days in the ICU were screened, pointing to a threshold in timing for the window to develop CMV in critical care setting. In a recent systematic review that included 13 studies (nine prospective and four retrospective), the incidence of CMV disease in critically ill patients, defined as the detection of antigenemia, DNAemia, or positive viral culture from blood samples with or without other clinical specimens, ranged from 0 to 36%. Notably, the reported incidence of the disease was much higher in studies that screened the patients weekly than those that screened only once within the first 4 days of admission to the ICU (6–33 vs. 0.8–1.2%), indicating that the disease happens frequently beyond the first 4 days post-admission. Among the studies using PCR detection (which is the most sensitive diagnostic test for CMV), the mean and median times of detection ranged from 4 to 12 days after ICU admission [8].

In summary, the incidence and demographics of CMV disease in the ICU were highly variable across studies, as a result of variation in study design and definition of the disease.

### Clinical manifestations

Identification of CMV disease in immunocompetent patients is complicated by its non-specific symptoms, multiorgan involvement, and the fact that its clinical manifestations converge with those of the critical illness. Heininger et al. [24] reported that serious organ involvement with CMV disease could occur in up to 10% of cases. A systematic review of studies reporting the clinical manifestations of severe CMV disease in immunocompetent ICU patients found that the gastrointestinal tract (hepatitis, gastroenteritis, duodenitis, enteritis, colitis, proctitis) is to be the most common, followed by central nervous system (encephalitis, myelitis, encephalomyelitis, meningitis, and meningioradiculopathy), and hematological system (hemolytic anemia, thrombocytopenia, disseminated intravascular coagulation, and pancytopenia) [35]. Other organs, such as the lungs and eyes, were rarely involved. CMV myocarditis, a recognized entity in immunocompromized patients, was not documented.

Vascular manifestations have been reported such as portal or femoropopliteal vascular thrombosis and pulmonary embolism that is thought to be related to vascular endothelial cells damage [35].

The immunomodulatory effects of CMV may be responsible for increased risk of secondary bacterial and fungal infections in critical care setting [3, 4].

CMV pneumonia is a well-known clinical manifestation of CMV disease in immunocompromized patients. However, lung involvement may be less recognizable in immunocompetent critically ill patients especially if they were intubated for other reasons, but few studies demonstrated that the prevalence of this disease in immunocompetent critically ill patients may be as high as 50% in patients with ventilator-associated pneumonia or ICU-acquired acute respiratory distress syndrome [36]. However, CMV disease is not necessarily the pathologic cause of ICU-acquired pneumonia and discrimination between a causal or associative relationship is challenging because the diagnosis depends on quality of the respiratory sample, pathologist skills, and variation of the diagnostic test. As an example of this variation, Coisel et al. [37] studied patients mechanically ventilated who are seropositive for CMV and found significant increased mortality in CMV-positive group; in addition, the diagnostic yield of BAL CMV PCR was 73% in comparison with the detection of CMV antigenemia which was 46%. Heininger et al. [38] studied patients admitted with severe sepsis of whom 31% developed ICU-acquired pneumonia and have shown slightly different diagnostic yield of tracheal aspirate CMV PCR of 70 versus 62% using blood CMV PCR. In a previous study, the same authors included only CMV surgical seropositive patients and found equal diagnostic yield between BAL PCR and blood PCR. Chiche showed different results, as the diagnostic yield of BAL was 26% (using CMV shell vial culture) compared to 85% using CMV antigenemia [26]. Papazian et al. [39] studied patients with ventilator-associated pneumonia and made histologic diagnosis of CMV pneumonia in 29% of the patients. The study found that BAL shell vial culture had a sensitivity/specificity of 53, 92% in comparison with histologic diagnosis. Ong et al. [40] studied immunocompetent patients with ARDS and found a CMV reactivation incidence of 27%, which was associated with significant increase in ICU mortality.

In general, CMV affects multiple organs at varying rates and severity. ICU-acquired pneumonia, especially VAP, appears to be a common CMV-associated disease that draw attention of ICU physicians and researchers.

### Diagnosis

Critically ill patients have dynamic immune system that affects the CMV detection window. The fact that immune suppression is not expected usually on presentation to intensive care, coupled with the ability of infected individuals to control the disease and eventually clear the virus at some point, affects the accuracy of diagnostic methods for CMV, all of that in addition to the differential sensitivities and clinical utilities of the techniques used to detect CMV (Table 3). In one major study assessing the rate of CMV disease in ICU patients, 50% of viremia was detected within the first 12 days of admission, but CMV infection was not detected earlier than the first 3 days [1].

**Table 3:** Diagnostic methods of CMV, advantages, and disadvantages

Diagnostic method	Advantages	Disadvantages
Anti-CMV immunoglobulins	Might be used for screening for latent CMV infection	Low sensitivity and specificity for active infection
CMV PCR assays	High sensitivity and specificity and considered gold standard, quick easy to perform, gives information of viral load, can be used for wide variety of samples	Better to be performed on whole blood, qualitative might be so sensitive and detect “innocent viral shedding” quantitative might be superior
CMV antigen assays	Quick and easy to perform, has comparable sensitivity and specificity to PCR	Might be inferior to PCR in case of leukopenia
Viral culture	Highly specific, can be performed on wide variety of samples	Time-consuming, low sensitivity
Histopathology	Highly specific, confirm CMV disease and pathogenicity and invasiveness	Invasive, low sensitivity, liable to sampling error, needs skilled pathologist and so operator dependent

Testing for CMV for the diagnosis of CMV disease should be based on predictors of CMV disease in addition to signs and other laboratory investigations directing toward viral origin of the current clinical status as indicators of liver and gastrointestinal involvement [41].

### Serology

The most commonly used tests to measure CMV-specific IgM or IgG levels are the enzyme-linked immune assays and anticomplement immunofluorescence assays. The presence of IgM antibodies indicates an acute CMV infection and can last for 4–6 months [42]. Because IgG seropositivity is long lasting, the measurement of IgG antibodies is not a reliable method to diagnose CMV infection. Critically ill patients may develop immune paralysis (anergy) that limits their ability to mount immune responses; therefore, it is important to note that negative serology does not exclude CMV infection. The main utility of serology testing at present is to screen for the potential of latent CMV reactivation [42].

### PCR

Due to its high sensitivity and rapid turnaround time, PCR is considered the gold standard method of diagnosing CMV infection [8]. Both qualitative and quantitative (viral load) PCR assays are available. However, quantitative tests are preferred as it quantifies viral load, which has prognostic importance [1]. Whole blood testing is more sensitive than plasma testing because it enables the detection of cell-free and intracellular viruses [43].

### CMV antigen assays

Antigen assays using immunofluorescent antibodies are quick and easy to perform and can be used to detect the CMV pp65 antigen in leukocytes [4]. In one study that compared PCR to antigen detection to diagnose CMV infection in critically ill patients, the antigen detection method was unable to identify 5 of the 11 CMV-positive patients detected by PCR. In addition, the diagnostic time of the PCR method was ahead than that of the antigen detection method [29]. However, meta-analyses performed by Osawa and Kalil revealed that the sensitivities and specificities of antigen detection methods are comparable to those of PCR detection methods [8, 44]. The only potential limitation of antigen assays is their low sensitivity in patients with leukopenia [5].

### Viral culture

Culture-based assays are not clinically used due to their low sensitivity and delay in receiving the results [8]. In a meta-analysis by Osawa and Singh [8], detection rate of CMV infection among studies using culture was 0–20% of CMV infections, whereas it was 0–32% using PCR and antigen assays. In a meta-analysis performed by Kalil and Florescu [34], the PCR/antigen detection methods achieved a CMV detection rate of 20%, whereas that the culture method was only 12%.

### Histopathology

Histopathology is the most specific method for diagnosing CMV, especially by detecting organ-specific manifestations related to CMV. However, this method is invasive and has a limited sensitivity that depends on the sampling site and pathologist’s skills.

We suggest using CMV PCR or CMV antigen detection assays to diagnose CMV disease not earlier than 3 days and up to 2 weeks after the onset of critical illness, especially when the pretest probability of the disease is high [1, 8].

### Prognosis

Studies addressing association of CMV disease and outcome in critically ill patients revealed inconsistent results.

A systematic review by Osawa and Singh [8] concluded that CMV disease in critically ill patients was associated with hepatic, respiratory, and renal dysfunctions; prolonged ICU stay, prolonged mechanical ventilation, increased incidence of bacterial and fungal infections, and increased mortality.

In a landmark prospective cohort study by Limaye et al. [1] of 120 CMV-seropositive critically ill patients, he found significant association between CMV disease and 30-day mortality and increased ICU length of stay. Notably high levels of viremia were associated with increased mortality and morbidity rates, as well as prolonged lengths of stay in the hospital, the latter of which increased with each log increase in the number of CMV copies (>1000 copies/ml up to 28 days of hospital length of stay). In addition, he observed higher rate of hospitalization beyond 30 days in CMV-infected patients indicating a long-term effect of CMV disease that persists beyond ICU discharge and even after clearance of the viremia. Of note, the study could not identify a relationship between the severity of critical illness (APACHE) and the risk of CMV disease, probably refuting the idea that CMV disease might be a marker of the underlying disease severity.

Regarding specific ICU population, a recent observational prospective study that included 86 CMV-seropositive septic patients found an association between CMV disease and longer ICU stay, prolonged mechanical ventilation, and impaired pulmonary gas exchange [38]. Coisel examined critically ill patients with ventilator-associated pneumonia and found a significant increase in ICU and 60-day mortality (50% CMV group vs. 20% in control group), longer ICU stay, and less ventilator-free days in patient with CMV disease [37].

On the other hand, a prospective observational study of 80 mechanically ventilated patients that were seropositive for CMV found no association between CMV disease and 28-day mortality, ICU mortality, ICU length of stay, or duration of mechanical ventilation, although the SOFA score was significantly higher in CMV-infected group [12]. Similar findings were also reported in a study that used CMV pp65 antigenemia as evidence of CMV disease [28].

These inconsistent findings are probably related to differences of studied populations, retrospective nature of several studies, differences in diagnostic tests, the unmeasured confounders; therefore, it is difficult to establish proved causality based on the existing studies, necessitating future studies with strict methodology in relation to blinding, studied population, testing timing, and method of testing preferably with tissue studies to clarify the causal relation between CMV disease and ICU mortality and morbidity.

In conclusion, although the current literature is inconsistent, there is strong evidence suggesting an association between CMV disease and higher morbidity and mortality in the ICU.

### Treatment and prevention

The central controversy of CMV disease in critically ill patients is whether to treat or not and whether treatment makes a difference to the patient’s outcomes or not.

Treatment of CMV disease as any other disease should be based on a benefit/risk ratio, taking into consideration all other factors associated with the diagnostic process and treatment such as invasiveness of the diagnostic method, side effects of anti-CMV medications which might be serious especially in critical ill patients, and risk of emergence of CMV resistance to the medications [45]. There is an agreement regarding treatment of established CMV disease and prophylactic treatment for certain populations of immunocompromized patients as is the case in organ transplant recipients, for whom the guidelines are well established [7, 44].

Similarly, the curative treatment of clinically confirmed CMV disease (detection of significant CMV viral load by PCR or antigen detection based on diagnostic method threshold, in addition to clinical condition attributed to CMV and ruling out other potential causes) in immunocompetent critically ill patients has been recommended, and support for such approach comes from studies like the one by Eddleston et al. [46] that examined the outcome of previously reported 34 cases of severe CMV disease in immunocompetent patients. It was found that multiorgan involvement was associated with poor outcome compared with isolated central nervous system involvement. Among patients with multiorgan involvement, 5 of 6 patients who received treatment of either ganciclovir or foscarnet recovered, while only 4 of 18 patients who did not receive either one of the above-mentioned drugs survived. The outcome noticed in this review pointed toward a potential benefit of antiviral treatment in this group of patients.

However, the benefit of preemptive treatment of subclinical detected viral replication with no proven direct organ pathology attributed to CMV, and the prophylactic treatment in immunocompetent patients deemed to be at risk of developing CMV disease is much less clear [45]. It is plausible to assume that the best strategy to handle CMV disease in the ICU is to prevent reactivation in seropositive patients as these are at high risk of developing the disease. At present randomized clinical trials examining the safety and efficacy of preventative strategies in critically ill patients are still going on.

Challenges for CMV preemptive and prophylactic treatment includes discouraging safety profiles of anti-CMV medications on the kidney and bone marrow especially in critically ill patients who already have organ dysfunction that puts them at extra risk of further dysfunctions and secondary infections (Table 4).

**Table 4:** Anti-CMV medications and common associated side effects

Agent	Mechanism of action	Common side effects
Ganciclovir	Competitively inhibits the binding of deoxyguanosine triphosphate to DNA polymerase resulting in inhibition of viral DNA synthesis	Thrombocytopenia, leukopenia, increased creatinine, fever, vomiting, diarrhea
Valganciclovir	Converted to ganciclovir in the body, much higher bioavailability of ganciclovir compared to oral ganciclovir	As ganciclovir
Foscarnet	Non-competitive inhibitor of many viral RNA and DNA polymerases	Electrolyte abnormalities, fever, vomiting, diarrhea, anemia, granulocytopenia, renal insufficiency, cardiotoxicity, central nervous system toxicity, hepatic toxicity
Cidofovir	Suppresses CMV replication by selective inhibition of viral DNA synthesis	Fever, alopecia, rash, ocular, renal, and gastrointestinal toxicity, cough, dyspnea

The other challenge is the detection of CMV, the current used methods, namely CMV PCR and CMV antigenemia, are highly accurate regarding sensitivity and specificity, but the challenge is that detection of CMV is highly sensitive to the timing and frequency of testing which is related to the window of emergence of CMV infection within the first 2 weeks of critical illness but not earlier than 3 days [8, 44]; secondly, it is unclear whether some cases represent innocent viral DNA or antigen shedding not necessarily related to tissue invasiveness and thus does not require therapy, which stress against random screening for CMV [13].

Papazian recommended in his recent review that treatment for evident CMV replication (blood or BAL significant viral load or antigen titer) is not indicated unless it is associated with lung infiltrates and at least two factors (prolonged mechanical ventilation, absence of bacterial agent, leukopenia, hemophagocytosis, high liver enzymes, hyperbilirubinemia, fever, or diarrhea) which if found points for CMV being a pathogen invading multiple organs and not only a bystander or innocent viral shedding [41].

So far no definite criterion is available to guide which critically ill patients should be screened for CMV disease. According to available literature certain populations as septic, burns, trauma, ICU-acquired pneumonia especially mechanically ventilated patients have been found at high risk of developing CMV disease across multiple studies [26, 41, 47]. Many authors recommended in these populations especially in case of prolonged mechanical ventilation with evidence of pneumonia, unexplained liver derangement or fever or unexplained digestive tract pathology, to screen for CMV disease and in case of CMV detection using PCR or antigenemia detection methods; then, treatment should be taken as benefit/risk ratio [37, 41, 48] (Table 4).

Regarding preemptive treatment, immunological assessment of IFN-γ produced by CMV-specific CD8+ T cells and NK cell function as mentioned in virulence section might be a strong trigger to start preemptive treatment for detected CMV, because there is evidence that impaired CMV-specific CD8+ T cells IFN-γ production prior to CMV reactivation predicts poor outcome, which favors treatment. This has not been applied clinically so far but might be promising in the future for challenging cases [10].

CMV might acquire resistance to the limited number of medications that are currently available; hence, their prophylactic use should be confined to confirmed cases or preemptive treatment until clear risk assessment tools for prophylactic therapy are developed in high-risk groups in the ICU [48].

## Conclusion

There are many uncertainties about CMV disease in the critically ill patients. First, while identification of the virus is common in critically ill patients, the actual rate of CMV as a disease in critically ill patients is unclear. Second, the importance of CMV detection in critically ill patients remains questionable, especially in the absence of histologic evidence of infection. On the other hand, the existing evidence is strong regarding the association between CMV detection and increased mortality and morbidity rates in ICU settings.

Still there are no high-quality data to guide the decision regarding when to treat detected CMV in ICU patients if there is no definite clinical confirmation of CMV disease (preemptive treatment), and there is no risk assessment tool to guide prophylactic therapy in ICU setting.

There is a need for well-conducted studies to develop risk assessment tool of CMV disease in critically ill patients, to estimate the real burden of CMV disease, and to examine the effect of prophylactic and preemptive treatment on morbidity and mortality. Potential answers to these questions will hopefully be answered by currently ongoing trials related to CMV in critical care. “Cytomegalovirus Control in Critical Care trial” (NCT01503918) is addressing the safety and success rate regarding preventing reactivation of latent cytomegalovirus infection in critically ill patients when treated with one of two different antiviral regimens: “Valaciclovir/Aciclovir or not.” “Study of Ganciclovir/Valganciclovir for Prevention of Cytomegalovirus Reactivation in Acute Injury of the Lung and Respiratory Failure” (NCT01335932) is addressing whether administration of ganciclovir in CMV-seropositive patients reduces serum IL-6 levels in immunocompetent adults with severe sepsis or trauma-associated respiratory failure based on the hypothesis that pulmonary and systemic CMV reactivation amplifies both lung and systemic inflammation mediated through specific cytokines. “Preemptive Treatment of Herpesviridae trial” (NCT02152358) is addressing whether preemptive treatment by ganciclovir (for positive CMV viremia) or aciclovir (for positive HSV oropharyngeal PCR) is able to increase the number of ventilator-free days at day 60 in ICU patients with prolonged mechanical ventilation having evident viral replication.

## References

[1] Limaye AP, Kirby KA, Rubenfeld GD, Leisenring WM, Bulger EM, Neff MJ (2008). Cytomegalovirus reactivation in critically ill immunocompetent patients. JAMA.

[2] Ziemann M, Sedemund-Adib B, Reiland P, Schmucker P, Hennig H (2008). Increased mortality in long-term intensive care patients with active cytomegalovirus infection. Crit Care Med.

[3] Sinclair J (2008). Human cytomegalovirus: latency and reactivation in the myeloid lineage. J Clin Virol.

[4] Ljungman P, Griffiths P, Paya C (2002). Definitions of cytomegalovirus infection and disease in transplant recipients. Clin Infect Dis.

[5] Kotton CN, Kumar D, Caliendo AM, Asberg A, Chou S, Danziger-Isakov L (2013). Updated international consensus guidelines on the management of cytomegalovirus in solid-organ transplantation. Transplantation.

[6] Andrews PA, Emery VC, Newstead C (2011). Summary of the British Transplantation Society guidelines for the prevention and management of CMV disease after solid organ transplantation. Transplantation.

[7] Tomblyn M, Chiller T, Einsele H, Gress R, Sepkowitz K, Storek J (2009). Guidelines for preventing infectious complications among hematopoietic cell transplant recipients: a global perspective. Preface. Bone Marrow Transplant.

[8] Osawa R, Singh N (2009). Cytomegalovirus infection in critically ill patients: a systematic review. Crit Care.

[9] Clari MA, Aguilar G, Benet I, Belda J, Gimenez E, Bravo D (2013). Evaluation of cytomegalovirus (CMV)-specific T-cell immunity for the assessment of the risk of active CMV infection in non-immunosuppressed surgical and trauma intensive care unit patients. J Med Virol.

[10] Chiche L, Forel JM, Thomas G, Farnarier C, Cognet C, Guervilly C (2012). Interferon-gamma production by natural killer cells and cytomegalovirus in critically ill patients. Crit Care Med.

[11] Venet F, Davin F, Guignant C, Larue A, Cazalis MA, Darbon R (2010). Early assessment of leukocyte alterations at diagnosis of septic shock. Shock.

[12] Frantzeskaki FG, Karampi ES, Kottaridi C, Alepaki M, Routsi C, Tzanela M (2015). Cytomegalovirus reactivation in a general, nonimmunosuppressed intensive care unit population: incidence, risk factors, associations with organ dysfunction, and inflammatory biomarkers. J Crit Care.

[13] Cook CH, Trgovcich J (2011). Cytomegalovirus reactivation in critically ill immunocompetent hosts: a decade of progress and remaining challenges. Antivir Res.

[14] Hummel M, Abecassis MM (2002). A model for reactivation of CMV from latency. J Clin Virol.

[15] Guedes MI, Risdahl JM, Wiseman B, Molitor TW (2004). Reactivation of porcine cytomegalovirus through allogeneic stimulation. J Clin Microbiol.

[16] Emery VC (2001). Progress in understanding cytomegalovirus drug resistance. J Clin Virol.

[17] Shuster EA, Beneke JS, Tegtmeier GE, Pearson GR, Gleaves CA, Wold AD (1985). Monoclonal antibody for rapid laboratory detection of cytomegalovirus infections: characterization and diagnostic application. Mayo Clin Proc.

[18] Penfold ME, Dairaghi DJ, Duke GM, Saederup N, Mocarski ES, Kemble GW (1999). Cytomegalovirus encodes a potent alpha chemokine. Proc Natl Acad Sci USA.

[19] Roberts ET, Haan MN, Dowd JB, Aiello AE (2010). Cytomegalovirus antibody levels, inflammation, and mortality among elderly Latinos over 9 years of follow-up. Am J Epidemiol.

[20] Sullivan V, Talarico CL, Stanat SC, Davis M, Coen DM, Biron KK (1992). A protein kinase homologue controls phosphorylation of ganciclovir in human cytomegalovirus-infected cells. Nature.

[21] Beersma MF, Bijlmakers MJ, Ploegh HL (1993). Human cytomegalovirus down-regulates HLA class I expression by reducing the stability of class I H chains. J Immunol.

[22] Littler E, Stuart AD, Chee MS (1992). Human cytomegalovirus UL97 open reading frame encodes a protein that phosphorylates the antiviral nucleoside analogue ganciclovir. Nature.

[23] Cook CH, Yenchar JK, Kraner TO, Davies EA, Ferguson RM (1998). Occult herpes family viruses may increase mortality in critically ill surgical patients. Am J Surg.

[24] Heininger A, Jahn G, Engel C, Notheisen T, Unertl K, Hamprecht K (2001). Human cytomegalovirus infections in nonimmunosuppressed critically ill patients. Crit Care Med.

[25] Prosch S, Wendt CE, Reinke P, Priemer C, Oppert M, Kruger DH (2000). A novel link between stress and human cytomegalovirus (HCMV) infection: sympathetic hyperactivity stimulates HCMV activation. Virology.

[26] Chiche L, Forel JM, Roch A, Guervilly C, Pauly V, Allardet-Servent J (2009). Active cytomegalovirus infection is common in mechanically ventilated medical intensive care unit patients. Crit Care Med.

[27] Cook CH, Martin LC, Yenchar JK, Lahm MC, McGuinness B, Davies EA (2003). Occult herpes family viral infections are endemic in critically ill surgical patients. Crit Care Med.

[28] Jaber S, Chanques G, Borry J, Souche B, Verdier R, Perrigault PF (2005). Cytomegalovirus infection in critically ill patients: associated factors and consequences. Chest.

[29] Kutza AS, Muhl E, Hackstein H, Kirchner H, Bein G (1998). High incidence of active cytomegalovirus infection among septic patients. Clin Infect Dis.

[30] von Muller L, Klemm A, Weiss M, Schneider M, Suger-Wiedeck H, Durmus N (2006). Active cytomegalovirus infection in patients with septic shock. Emerg Infect Dis.

[31] Domart Y, Trouillet JL, Fagon JY, Chastre J, Brun-Vezinet F, Gibert C (1990). Incidence and morbidity of cytomegaloviral infection in patients with mediastinitis following cardiac surgery. Chest.

[32] Soderberg-Naucler C, Fish KN, Nelson JA (1997). Reactivation of latent human cytomegalovirus by allogeneic stimulation of blood cells from healthy donors. Cell.

[33] Bowden RA, Sayers M, Flournoy N, Newton B, Banaji M, Thomas ED (1986). Cytomegalovirus immune globulin and seronegative blood products to prevent primary cytomegalovirus infection after marrow transplantation. N Engl J Med.

[34] Kalil AC, Florescu DF (2009). Prevalence and mortality associated with cytomegalovirus infection in nonimmunosuppressed patients in the intensive care unit. Crit Care Med.

[35] Rafailidis PI, Mourtzoukou EG, Varbobitis IC, Falagas ME (2008). Severe cytomegalovirus infection in apparently immunocompetent patients: a systematic review. Virol J.

[36] Papazian L, Doddoli C, Chetaille B, Gernez Y, Thirion X, Roch A (2007). A contributive result of open-lung biopsy improves survival in acute respiratory distress syndrome patients. Crit Care Med.

[37] Coisel Y, Bousbia S, Forel JM, Hraiech S, Lascola B, Roch A (2012). Cytomegalovirus and herpes simplex virus effect on the prognosis of mechanically ventilated patients suspected to have ventilator-associated pneumonia. PLoS One.

[38] Heininger A, Haeberle H, Fischer I, Beck R, Riessen R, Rohde F (2011). Cytomegalovirus reactivation and associated outcome of critically ill patients with severe sepsis. Crit Care.

[39] Papazian L, Fraisse A, Garbe L, Zandotti C, Thomas P, Saux P (1996). Cytomegalovirus. An unexpected cause of ventilator-associated pneumonia. Anesthesiology.

[40] Ong DS, Spitoni C, Klein Klouwenberg PM, Verduyn Lunel FM, Frencken JF, Schultz MJ (2016). Cytomegalovirus reactivation and mortality in patients with acute respiratory distress syndrome. Intensive Care Med.

[41] Papazian L, Hraiech S, Lehingue S, Roch A, Chiche L, Wiramus S (2016). Cytomegalovirus reactivation in ICU patients. Intensive Care Med.

[42] Chou S (1990). Newer methods for diagnosis of cytomegalovirus infection. Rev Infect Dis.

[43] Lisboa LF, Asberg A, Kumar D, Pang X, Hartmann A, Preiksaitis JK (2011). The clinical utility of whole blood versus plasma cytomegalovirus viral load assays for monitoring therapeutic response. Transplantation.

[44] Kalil AC, Levitsky J, Lyden E, Stoner J, Freifeld AG (2005). Meta-analysis: the efficacy of strategies to prevent organ disease by cytomegalovirus in solid organ transplant recipients. Ann Intern Med.

[45] Chanques G, Jaber S (2014). Treating HSV and CMV reactivations in critically ill patients who are not immunocompromised: con. Intensive Care Med.

[46] Eddleston M, Peacock S, Juniper M, Warrell DA (1997). Severe cytomegalovirus infection in immunocompetent patients. Clin Infect Dis.

[47] Kalil AC, Florescu DF (2011). Is cytomegalovirus reactivation increasing the mortality of patients with severe sepsis?. Crit Care.

[48] Forel JM, Martin-Loeches I, Luyt CE (2014). Treating HSV and CMV reactivations in critically ill patients who are not immunocompromised: pro. Intensive Care Med.

[49] Florescu DF, Kalil AC (2011). Cytomegalovirus infections in non-immunocompromised and immunocompromised patients in the intensive care unit. Infect Disord Drug Targets.

[50] Docke WD, Prosch S, Fietze E, Kimel V, Zuckermann H, Klug C (1994). Cytomegalovirus reactivation and tumour necrosis factor. Lancet.

[51] Stephan F, Meharzi D, Ricci S, Fajac A, Clergue F, Bernaudin JF (1996). Evaluation by polymerase chain reaction of cytomegalovirus reactivation in intensive care patients under mechanical ventilation. Intensive Care Med.

[52] Razonable RR, Fanning C, Brown RA, Espy MJ, Rivero A, Wilson J (2002). Selective reactivation of human herpesvirus 6 variant A occurs in critically ill immunocompetent hosts. J Infect Dis.

[53] von Muller L, Klemm A, Durmus N, Weiss M, Suger-Wiedeck H, Schneider M (2007). Cellular immunity and active human cytomegalovirus infection in patients with septic shock. J Infect Dis.

[54] Chilet M, Aguilar G, Benet I, Belda J, Tormo N, Carbonell JA (2010). Virological and immunological features of active cytomegalovirus infection in nonimmunosuppressed patients in a surgical and trauma intensive care unit. J Med Virol.

[55] Smith CA, Conroy LT, Pollock M, Ruddy J, Binning A, McCruden EA (2010). Detection of herpes viruses in respiratory secretions of patients undergoing artificial ventilation. J Med Virol.

[56] Bordes J, Maslin J, Prunet B, d’Aranda E, Lacroix G, Goutorbe P (2011). Cytomegalovirus infection in severe burn patients monitoring by real-time polymerase chain reaction: a prospective study. Burns.

[57] De Vlieger G, Meersseman W, Lagrou K, Wouters P, Wilmer A, Peetermans WE (2012). Cytomegalovirus serostatus and outcome in nonimmunocompromised critically ill patients. Crit Care Med.

[58] Ishioka H, Sanui M, Tsutsumi Y, Yanase F, Shiotsuka J (2014). Low prevalence of active cytomegalovirus infection in a cardiovascular intensive care unit. J Intensive Care.

[59] Walton AH, Muenzer JT, Rasche D, Boomer JS, Sato B, Brownstein BH (2014). Reactivation of multiple viruses in patients with sepsis. PLoS One.

[60] Ong DS, Klein Klouwenberg PM, Verduyn Lunel FM, Spitoni C, Frencken JF, Dekker HA (2015). Cytomegalovirus seroprevalence as a risk factor for poor outcome in acute respiratory distress syndrome. Crit Care Med.

